# Harms from Other People’s Gambling: Associations with an Individual’s Own Gambling Behaviours, Health Risk Behaviours, Financial Problems, General Health, and Mental Wellbeing

**DOI:** 10.1007/s10899-024-10291-w

**Published:** 2024-03-15

**Authors:** Charley Wilson, Nadia Butler, Zara Quigg

**Affiliations:** Public Health Institute, Liverpool John Moores University, 3rd Floor Exchange Station, Liverpool, L3 2ET UK

**Keywords:** Gambling, Affected others, Health risk behaviours, Wellbeing

## Abstract

This study aimed to explore relationships between being an ‘affected other’ (AO) and an individual’s own gambling, health risk behaviours, financial problems, poor general health, and low mental wellbeing. A cross-sectional survey using representative and supplementary convenience samples was undertaken with 1234 residents of a British Island aged 16 + years. Being an AO was measured as having a partner or relative who has been gambling regularly in the past 12 months. PGSI was used to assess gambling severity. Health risk behaviours included: poor diet, low physical activity, daily smoking, and binge drinking. Other measures included experiencing financial problems, poor general health, and low mental wellbeing. Analyses were performed using χ^2^ and binary logistic regressions. 11.0% of participants were AOs. AOs were more likely to gamble at at-risk/problem-gambling levels and experience financial problems compared to those who were not affected others. The significant relationship between being an AO and low mental wellbeing was mediated by experiencing financial problems. The relationship between being an AO and engaging in two or more health risk behaviours was no longer significant after controlling for sociodemographics and an individual’s own gambling. The relationship between being an AO and poor general health was no longer significant after controlling for sociodemographics, health risk behaviours and an individual’s own gambling. AOs experienced risks to health and wellbeing, with findings not limited to AOs specifically with a relationship to an individual with problem-gambling. Therefore, support for AOs should be more widely available, aiming to address AOs’ needs holistically.

**Supplementary Information:**

The online version contains supplementary material available at 10.1007/s10899-024-10291-w.

## Introduction

Globally, gambling is increasingly recognised as a public health concern with different countries putting different levels of regulations in place to prevent and respond to gambling harms. Work has also taken place in different countries to understand the proportion of the population experiencing gambling harms. In 2022/23, the prevalence of problem gambling in the United Kingdom’s (UK) adult population was estimated to be 0.3%, with a further 3.0% at low or moderate risk (The Gambling Commission, 2023). Other data has found such rates to be higher in the UK, with Gunstone et al. (2021) finding the prevalence of problem gambling to be 2.8%, with a further 9.9% at low or moderate risk. Differences between prevalence rates across studies are heavily impacted by the methods and measures used. The study by Gunstone et al. (2021) is considered more reliable due to the larger sample size (n = 18,038), and the full Problem Gambling Severity Index (PGSI) tool being utilised, compared to The Gambling Commission (2023) data which had a smaller sample size (n = 4002) and used the short form PGSI (Newall et al., 2022; Sturgis, 2020; Sulkunen et al., 2019; Wardle, 2015). Nonetheless, while such figures may be relatively small percentages, this would amount to a large problem across the population, with over 1.4 million people considered to have problem gambling in the UK (Gunstone et al., 2021). While harms at the problem gambling level are significant, the burden of harm is not solely isolated to this group. Research has demonstrated that the majority of gambling harms fall on those below the threshold for problem gambling, indicating that gambling is a public health issue impacting a greater number than those with problem gambling alone (Browne, 2020; Browne et al., 2017; Canale et al., 2016).

Globally, research has demonstrated a number of gambling-related harms spanning individual, relationship, community and societal levels (Langham et al., 2015; Orford, 2020). Harms to the individual have been demonstrated, ranging from lower-level harms such as lost time and smaller financial problems to serious harms with long-term implications including debt, crime, homelessness, and suicide (Langham et al., 2015; Orford, 2020; Wardle et al., 2020). Furthermore, a representative survey study from a British Island (n = 2303) showed that compared to those with non-problem gambling, those gambling at low- and moderate-risk and problem gambling levels were more likely to have a poor diet, smoke tobacco, and have harmful alcohol use, as well as having increased likelihood of experiencing poorer general health and low mental wellbeing (Butler et al. 2019).

A less well-researched area of gambling-related harm is the impacts that gambling has on individuals who have a significant relationship with a person with problem gambling (e.g., children, partners etc.) termed as ‘affected others’ (AOs). Research has tried to estimate the prevalence of AOs in the general population and has shown that problem gambling impacts a greater proportion of the population than the gambling individual alone (Tulloch et al., 2021). In the UK, Gunstone et al. (2021) found that 6.5% of the UK population were individuals who ‘knew someone with a gambling problem (either currently, or in the past) and feel that they have personally experienced negative effects from this person’s gambling behaviour’. Further, research from Australia has estimated that for each individual with problem gambling, their gambling affects between four and six other individuals, while those with moderate-risk and low-risk gambling affect one and three others respectively (Goodwin et al., 2017).

Research has shown that there is a large degree of cross-over in the experiences of gambling-related harms by the gambling individual and by AOs (Li et al., 2017). Harms to AOs impact in numerous ways, including financial problems, reduced emotional and psychological wellbeing, harm to physical health, and significant breakdowns in the relationship with the individual who gambles, characterised by a lack of trust and interpersonal conflicts, which can include violence (Banks et al., 2018; Castren et al., 2021; Dowling et al., 2021; Gunstone et al., 2021; Holdsworth et al., 2013; Langham et al., 2015; Lind et al., 2022; Riley et al., 2018; Subramaniam et al., 2017; Tulloch et al., 2021; Velleman et al., 2015). Research has also illustrated that AOs were more likely to engage in health risk behaviours including harmful alcohol use, daily tobacco smoking, over-eating, as well as risky gambling behaviours themselves (Dowling et al., 2021; Lind et al., 2022; Riley et al., 2018).

When examining harms to AOs there has been limited use of validated measures or scales across factors such as mental wellbeing (Dowling et al., 2021; Tulloch et al., 2021). Studies examining gambling-related harms to AOs have utilised different measures for defining AOs, with some studies covering family members only, others including friends and colleagues. Some studies have included only those who are currently AOs, while other studies include those who have ever been an AO. However, one factor most studies of AOs have in common is that they tend to include AOs on the basis that they have a relationship with somebody specifically with problem gambling. While there is a validated measure for identifying individuals who may have problem gambling, or experience sub-problem gambling level harms, no such measure exists for identifying AOs. Therefore, a range of experiences of gambling-related harms may be missed in AOs with a relationship to somebody who gambles at sub-problem gambling levels (Dowling et al., 2021; Tulloch et al., 2021).

Most research on AOs has not examined harms occurring at the sub-problem gambling level. Further, only a small number of studies have examined harms to AOs in general population samples. Across both of these areas the evidence base is particularly scarce from a British perspective. The current study aims to address this gap, in a general population sample from a British Island, by utilising a measure for identifying AOs which did not depend solely on the AO having a relationship with someone who gambles at the problem gambling level. Specifically, the current study aims to explore relationships between an individual’s own gambling behaviours, health risk behaviours, financial problems, poor general health, low mental wellbeing, and having a relative or partner who has been gambling regularly in the past 12 months.

## Methods

The sample included individuals residing on a British Island with a population of 53,627 residents aged 16 + years (86.1% of all residents). 24.7% of all residents were aged 16–34 years, 28.0% were aged 35–54, and 33.4% of residents were aged 55 + . There was a relatively even split of males (49.2%) and females (50.8%) aged 16 + living on the British Island.

There are a number of online and offline gambling environments and opportunities available on the British Island, residents can participate in gambling activities including lotteries, raffles, fixed-odds betting terminals, online gambling (such as for sports gambling and online bingo), and scratch cards, there are also several betting shops and bookmakers available.

### Study Design and Participants

A cross-sectional survey of residents aged 16 + was undertaken between October and November 2019. The survey was conducted in two phases. Phase 1 was an invited representative sample of the British Island’s population, with 7000 addresses selected. A random sample was then selected, stratified by parish and social or non-social housing (due to a low expected response rate amongst individuals in social housing). Following phase 1, phase 2 was implemented, consisting of a convenience sample, making the survey open-access to all members of the public, promoted through media channels.

The total sample size was n = 1234, equating to 2.4% of the population (aged 16 + years).

### Measures

#### Affected Others

A self-report item was included asking participants: ‘In the last 12 months, has your partner or one of your relatives been gambling regularly?’ Response options included: Yes; No; or, Don’t know. AOs were coded as those who responded yes; zero participants responded don’t know. Those who answered ‘Yes’ to this question were also asked whether they experienced several harms directly as a result of their partner or relative’s gambling.

#### Gambling Behaviours and Problem Gambling

Problem and at-risk gambling was measured using the PGSI. The PGSI is a self-report, validated instrument for use in general populations (Ferris & Wynne, 2001; Holtgraves, 2008). The PGSI tool consists of nine questions, each measured on a four-point scale (0 = never, 1 = sometimes, 2 = most of the time, 3 = almost always). Scores for each question are summed, giving a total overall score ranging from 0 to 27, with higher scores indicating a greater severity of gambling risk. PGSI score can be divided into the following categories: 0 = non-problem gambling; 1–2 = low-risk gambling; 3–7 = moderate-risk gambling; 8 +  = problem gambling. Due to small sample sizes, the low- and moderate-risk and problem gambling categories were combined to create a dichotomous variable of non-problem gambling and at-risk/problem gambling. Other gambling behaviours such as the frequency of participation in different gambling activities, and whether individuals gambled online were also measured.

#### Health Risk Behaviours

Four measures of health risk behaviours were included for analyses: poor diet, low physical activity, daily smoking, and binge drinking. Poor diet was defined as eating less than 2 portions of fruit and vegetables (excluding potatoes) per day. Low physical activity was defined as taking part in less than 150 min of physical activity that was ‘enough to raise your breathing rate’ in the previous week. Daily smoking was defined as the current smoking of tobacco on a daily basis. Consumption of alcohol was measured using AUDIT-C questions. AUDIT-C is a commonly used validated screening tool for identifying harmful alcohol use (Bush et al., 1998). Binge drinking was defined as having 6 or more standard drinks (one standard drink in the UK is equal to one UK unit of alcohol (10mls of pure alcohol)) on one occasion, at least once per week. Engaging in two or more health risk behaviours was defined as engaging in two or more of having a poor diet, low physical activity, daily smoking, and binge drinking.

#### Financial Problems

Experiencing financial problems was defined as those who answered Yes to the question ‘In the past 12 months, have you been behind (e.g., paid late, had to borrow money, or have gone without) with payments for expenses such as rent, utilities, mortgage repayments, taxes etc.?’

#### Poor General Health

General health was measured using a self-reported question on ‘health today’, this question asked individuals to rate their health on a visual scale from 0–10 (0 indicating ‘the worst health you can imagine’ and 10 indicating ‘the best health you can imagine’). Poor general health was defined as scores of 5 or less.

#### Low Mental Wellbeing

Mental wellbeing was measured using the Short Warwick–Edinburgh Mental Wellbeing Scale (SWEMWBS; Stewart-Brown et al., 2009). This is a validated scale including seven items about an individual’s current mental wellbeing, scored on a 5-point scale (1 = none of the time; 2 = rarely; 3 = some of the time; 4 = often; 5 = all of the time). Total scores on the SWEMWBS range from 7 to 35, with higher scores indicating higher levels of mental wellbeing. Raw scores are then converted to metric scores using a standard conversion table (Stewart-Brown et al., 2009). Scores were dichotomised to indicate low mental wellbeing as more than one standard deviation (4.397) below the mean (24.378), thus low mental wellbeing was operationalised as scores of < 19.981.

#### Sociodemographic Factors

Measured sociodemographic factors included: sex, age (16–34; 35–54; 55 + years), and income level (< £20,000; £20,000–£79,999; £80,000 +).

#### Analyses

Analyses were undertaken in SPSS (v.28). Bivariate analyses using χ^2^ tests were used to illustrate significant associations between AO status and sociodemographics, an individual’s own gambling behaviours, health risk behaviours, financial problems, poor general health, and low mental wellbeing. Binary logistic regression (enter method) models were used to estimate the size and significance of associations between AO status and each measure of interest, after controlling for sociodemographics. In regressions for health risk behaviours and financial problems, the individual’s own gambling behaviours were additionally controlled for within the same model. In the regressions for both poor general health and low mental wellbeing, the individual’s own gambling behaviours and all health risk behaviours were additionally controlled for in a separate model. In the regressions for low mental wellbeing experiencing financial problems was additionally controlled for in another separate model. Financial problems were not included in models examining the relationship between AO status and poor general health, as this relationship was no longer significant once health risk behaviours and the individual’s own gambling were controlled for.

### Results

#### Affected Others’ Sociodemographics

Just over one in ten (11.0%; n = 126) study participants reported that they had a partner or relative who had been gambling regularly in the past 12 months (i.e. AO). 33.6% (n = 41) of AOs indicated that it was their spouse or partner who was gambling regularly, 13.9% (n = 17) their sibling, 9.0% (n = 11) their parent, 36.9% (n = 45) another relative, 6.6% (n = 8) had multiple relatives who were gambling regularly in the past year. Table 1 shows the sociodemographic characteristics of AOs. In bivariate analyses, there were significant associations between AO status and age (16–34, 17.6%; 35–54, 11.9%; 55 + , 7.9%; *p* < 0.001). There were no significant relationships between AO status and sex or income level.

**Table 1:** Sociodemographics of affected others

	All % (n)	Affected others % (n)	Non-affected others % (n)	χ^2^	Significance (*p*)
Overall	–	11.0 (126)	89.0 (1020)	–	–
*Sex*					
Male	45.9 (561)	9.7 (51)	90.3 (477)	–	–
Female	54.1 (661)	11.7 (71)	88.3 (537)	1.201	NS
*Age*					
16–34	19.4 (240)	17.6 (39)	82.4 (183)	–	–
35–54	29.9 (369)	11.9 (41)	88.1 (303)	–	–
55 +	50.6 (625)	7.9 (46)	92.1 (534)	15.664	< 0.001
*Income level*					
< £20,000	9.1 (96)	13.6 (11)	86.4 (70)	–	–
£20,000–79,999	59.5 (630)	10.1 (59)	89.9 (525)	–	–
£80,000 +	31.4 (332)	12.2 (39)	87.8 (281)	1.480	NS

#### Affected Others’ Experience of Gambling-Related Harms

AOs reported experiencing several harms as a direct result of their relative or partner’s gambling. Overall, 19.5% of AOs experienced one or more harms directly related to their relative or partner’s gambling. 12.9% reported that they had a serious argument not including violence, or had been emotionally hurt or neglected by the individual who had been gambling. 8.9% of AOs reported that they missed out on money that would have improved their quality of life, as it was spent on gambling activities. 8.9% also reported being let down by the gambling individual who failed to do something they were counting on them to do because of their gambling. 5.6% of AOs had something broken, damaged, or stolen by the gambling individual. 4.8% of AOs had to stop seeing or being in contact with the other individual due to their gambling. 2.4% felt physically threatened, physically hurt due to assault or violence, or concerned that the individual who had been gambling regularly may cause harm to their children. There were no significant associations between experiencing these gambling-related harms and sociodemographics.

#### Affected Others’ Gambling Behaviours

AOs gambled on a range of activities within the past 12 months (lotteries 81.0%; scratch cards 59.5%; horse and dog race betting 28.6%; sports betting 22.4%; private betting 21.1%; bingo 13.5%; fruit or slot machines 7.1%; online fruit machines/instant win games 5.6%; roulette, cards or dice 4.0%; poker 2.4%). AOs were significantly more likely to have gambled on any gambling activity and to have gambled online in the past 12 months than NAOs (Table 2). There was also a significant association between AO status and gambling frequency, with a higher proportion of AOs gambling at least once a week compared to NAOs (Table 2).

**Table 2:** Affected others’ gambling behaviours

	All %(n)	Affected others % (n)	Non-affected others % (n)	χ^2^	Significance (*p*)
Any past year gambling	78.5 (969)	87.3 (110)	77.7 (793)	6.130	< 0.05
Any online gambling	12.7 (146)	24.3 (28)	11.4 (109)	15.380	< 0.001
*Gambling frequency*					
Never	25.1 (265)	14.3 (16)	26.1 (227)		
Less than once a month/at least once a month	61.3 (648)	55.4 (62)	62.6 (544)		
At least once a week	13.6 (144)	30.4 (34)	11.3 (98)	33.296	< 0.001
*Gambling risk level*					
Non-problem gambling	94.4 (1154)	88.0 (110)	95.4 (968)		
At-risk/problem gambling	5.6 (68)	12.0 (15)	4.6 (47)	11.753	< 0.001

There was a significant association between AO status and at-risk/problem gambling. After controlling for sex, age, and income level, AOs were 2.2 times (AOR = 2.17 (1.08–4.38); *p* < 0.05) more likely to gamble at at-risk/problem gambling levels themselves, compared to NAOs.

#### Affected Others’ Health Risk Behaviours

Table 3 describes the relationships between being an AO and health risk behaviours. In bivariate analyses, the prevalence of all health risk behaviours was higher amongst AOs when compared to NAOs. Further, associations with poor diet and low physical activity were significant, however, associations with binge drinking, and daily smoking were not. There was also a significant relationship between AO status and engaging in two or more health risk behaviours. After controlling for sex, age, income level, and an individual’s at-risk/problem gambling the associations between AO status and poor diet, low physical activity, and engaging in two or more health risk behaviours were no longer significant (Table 4; Supplementary Tables 1, 2 and 5).

**Table 3:** Associations between affected other status and health risk behaviours, financial problems, general health, and mental wellbeing

	All % (n)	Affected others % (n)	Non-affected others % (n)	χ^2^	Significance (*p*)
Poor diet	8.9 (109)	15.2 (19)	7.8 (79)	7.861	< 0.01
Low physical activity	33.3 (403)	43.1 (53)	32.1 (322)	5.991	< 0.05
Daily smoking	6.4 (79)	9.5 (12)	5.5 (56)	3.199	NS
Binge drinking	15.6 (169)	20.7 (23)	14.9 (134)	2.545	NS
2 or more health risk behaviours	12.5 (133)	19.4 (21)	11.3 (100)	5.894	< 0.05
At-risk/problem gambling	5.6 (68)	12.0 (15)	4.6 (47)	11.753	< 0.001
Financial problems	8.2 (101)	17.6 (22)	6.4 (65)	19.872	< 0.001
Poor general health	13.6 (168)	19.0 (24)	11.9 (121)	5.174	< 0.05
Low mental wellbeing	17.7 (215)	30.2 (38)	15.1 (152)	18.055	< 0.001

**Table 4:** Multivariate relationships between health risk behaviours, financial problems, general health, and mental wellbeing, and affected other status

	Affected others AOR (95% CI)	Significance (*p*)
Poor diet	1.48 (0.75–2.91)	0.255
Low physical activity	1.29 (0.84–1.98)	0.242
Daily smoking	1.55 (0.73–3.27)	0.253
Binge drinking	1.65 (0.96–2.85)	0.070
2 or more health risk behaviours	1.75 (0.97–3.17)	0.065
Financial problems	2.12 (1.10–4.07)	0.025
Poor general health	1.85 (0.99–3.47)	0.055
Low mental wellbeing	1.75 (1.02–2.99)	0.042

#### Affected Others’ Financial Problems

In bivariate analyses, the prevalence of experiencing financial problems was higher amongst AOs when compared to NAOs (Table 3). The relationship between experiencing financial problems and AO status was significant. After controlling for sex, age, income level, and an individual’s at-risk/problem gambling AOs were 2.1 times (AOR = 2.12 (1.10–4.07); *p* < 0.05) more likely to experience financial problems than NAOs (Table 4; Supplementary Table 6).

#### Affected Others’ General Health and Mental Wellbeing

In bivariate analysis, the prevalence of both poor general health and low mental wellbeing was significantly higher in AOs, compared to NAOs (Table 3). In multivariate analyses, after controlling for sex, age, and income level, AOs were 1.8 times more likely (AOR = 1.82 (1.06–3.13); *p* < 0.05) to have poor general health, and 1.7 times more likely (AOR = 1.70 (1.04–2.77); *p* < 0.05) to have low mental wellbeing, compared to NAOs (Supplementary Tables 7 and 8). After additionally controlling for an individual’s own at-risk/problem gambling, and all health risk behaviours, AOs remained 1.7 times more likely to have low mental wellbeing than NAOs, however, the relationship between AO status and poor general health was no longer significant (Table 4; Supplementary Tables 7 and 8). After additionally controlling for financial problems, the relationship between AO status and low mental wellbeing was no longer significant (AOR = 1.65; CIs = 0.95–2.87; Supplementary Table 8).

### Discussion

Findings in this study demonstrate links between being an AO and increased engagement in riskier gambling behaviours and experiencing financial problems. This study found that an individual’s own gambling behaviours mediated relationships between being an AO and increased engagement in health risk behaviours. The relationship between AO status and poor general health was mediated by an individual’s own gambling behaviours and engagement in health risk behaviours. The significant relationship between AO status and low mental wellbeing was not initially mediated by an individual’s own gambling behaviours or engagement in health risk behaviours. However, after additionally controlling for experiencing financial problems this relationship was no longer significant. Crucially, this study indicated that such outcomes are not limited to individuals with relationships specifically with someone with problem gambling, but incorporated relationships with someone who gambles at any level (but at least regularly).

#### Relationships with Own Gambling

The finding in the current study that at-risk/problem gambling was over twice as likely among AOs compared to NAOs mirrors results seen in a Finnish population study (Lind et al., 2022). This relationship is likely seen due to social and environmental factors, which promote gambling as an increasingly normalised and shared activity between family members and peers, with gambling marketing playing a key role in this (Guillou-Landreat et al., 2021; King et al., 2010; Subramaniam et al., 2017). This relationship is further supported by research from Australia, which has demonstrated that within the social circles of those with at-risk/problem gambling there is an increased prevalence of gambling participation and harms (Russell et al., 2018). Such social circles were found to be deeply interconnected, and both influenced and normalised gambling behaviours (Russell et al., 2018). Family gambling attitudes and behaviours are a key factor that shape an individual’s gambling behaviours, both in youth and as an individual progresses through the life-course, with parental gambling heavily linked to the gambling behaviours of adolescents, including early initiation (Reith and Dobbie 2011; Emond & Griffiths, 2020). Certain family norms and practices may promote gambling as a normalised and shared family activity, however, also promote risks of experiencing future gambling harm. For example, in the UK, family members may purchase scratch cards for children, or family holidays at seaside resorts may involve children using ‘legal youth gambling products’ such as coin pusher machines, however, use of such products is also associated with future adult gambling participation and harm (Emond & Griffiths, 2020; Newall et al., 2021). Such interactions between family and youth gambling may play a role in shaping an individual’s future gambling activities, and reinforce relationships between individuals who gamble, which may help to explain at least a part of the relationship seen in the current study between being an AO and at-risk/problem gambling (Reith & Dobbie, 2011). This is particularly interesting in the context of the finding in the current study showing that for nearly one in ten AOs it was their parent who had been gambling regularly.

The links between being an AO and an individual’s own experiences of at-risk and problem gambling are concerning, as the relationships between these individuals may exacerbate difficulties in reducing gambling consumption, socially reinforcing experiences of gambling harm (Russell et al., 2018). Further, this relationship is likely to make situations whereby both individuals are proximal to each other in their daily lives increasingly difficult, as there will be a dual burden of experiencing harm not only from an individual’s own gambling behaviours but also the gambling behaviours of the other individual.

#### Relationships with Health Risk Behaviours

In contrast to other studies of AOs, there were no significant associations between AO status and any individual health risk behaviour. Methodological differences may partly explain this, in that the measure for being an AO in the current study was not exclusive to those with a relationship to someone with problem gambling. Therefore, for a higher proportion of individuals in the current study, the other individual’s gambling may not yet be significant enough of a problem to influence individual health risk behaviours, particularly not as a coping mechanism (Banks et al., 2018). However, an individual’s own gambling mediated the relationship between AO status and engagement in two or more health risk behaviours, so that this relationship was no longer significant. It is possible that through increased participation in gambling, AOs may be increasingly exposed to a range of health risk behaviours more generally, thus increasing engagement with health risk behaviours overall. This is supported by prior research by Butler et al. (2019) which demonstrated that individuals were more likely to engage in a range of health risk behaviours across the spectrum of gambling risk levels. Further Butler et al. (2019) suggested that health risk behaviours may be linked through environments and social norms, which provide opportunities to engage in multiple health risk behaviours in one setting. Any relationship which shows increased engagement in two or more health risk behaviours (even if mediated by an individual’s own gambling behaviours) warrants concern, as the cumulative effects of engaging in more than one health risk behaviour are greater than heightened engagement in one health risk behaviour alone, increasing the risks of morbidity (Bellis et al., 2016).

Findings also demonstrated direct harms from the other individual’s gambling, with one in five AOs experiencing at least one harm as a direct result of their relative or partner’s gambling. Conflicts in the relationship were the most prevalent harms shown, with these types of harms present for over one in ten AOs. A small number of AOs reported that they had experienced violence (or the threat thereof) or were concerned that the individual who had been gambling may harm their children. Previous literature has documented the impacts of gambling on violent outcomes in domestic settings, both in terms of intimate partner violence and child maltreatment (Hing et al., 2022; Roberts et al., 2016). However, such outcomes have not been well explored at the sub-problem gambling level, or outside of a domestic setting. It is beyond the scope of the current study to discern whether these outcomes were limited to those with a relationship to an individual with problem gambling or not. Future research may wish to prioritise exploring violent outcomes related to gambling, both at the sub-problem gambling level and for AOs outside of the same household.

#### Relationships with Experiencing Financial Problems

The current study found significant associations between AO status and experiencing financial problems. Further, it was also found in the current study that nearly one in ten AOs experience financial harm as a direct result of their partner or relative spending money on gambling activities. These findings are consistent with previous research showing the significant burden of financial problems on people experiencing problem gambling and AOs, which often reach crisis points (Banks et al., 2018; Dowling et al., 2021; Langham et al., 2015). Research has shown that financial problems are one of the first presenting gambling-related harms that AOs become aware of, with financial problems often preceding further second-hand harms such as emotional stressors, conflicts, and breakdown of relationships (Dowling et al., 2021; Holdsworth et al., 2013; Subramaniam et al., 2017). Prior research has demonstrated the direct detrimental impact of financial problems, such as unsecured debt, on mental health and wellbeing (Brown et al., 2005; Kiely et al., 2015; Richardson et al., 2013). The processes of dealing with financial problems can also be detrimental to health and wellbeing through, for example, having to work extra hours, having to substitute healthier choices (e.g., food) for cheaper less healthy alternatives, or go without things which are beneficial to health (e.g., a gym membership) (Banks et al., 2018). Financial problems also have significant implications for longer-term harms from gambling, even after an individual may cease gambling behaviours, with ongoing financial hardships, poverty, and reliance on welfare having notable impacts on health and wellbeing, with such harms also having intergenerational impacts (Dowling et al., 2021; Langham et al., 2015).

#### Relationships with Poor General Health and Low Mental Wellbeing

Associations between AO status and poor general health and low mental wellbeing were found in the current study. However, controlling for health risk behaviours and an individual’s own gambling behaviour mediated the relationship between being an AO and poor general health, with this association no longer significant. It is possible that through increased engagement with riskier gambling and other health risk behaviours (linked through gambling environments and social processes which promote engagement in multiple health risk behaviours), being an AO may increase risks of experiencing poor general health.

When an individual’s own gambling behaviour and health risk behaviours were controlled for, the relationship between AO status and low mental wellbeing remained significant, however, once financial problems were additionally controlled for this relationship was no longer significant. This indicates that for AOs, increased experiences of financial problems may be a driving cause of low mental wellbeing in this group. This is interesting considering the measure for being an AO in this study may include a wider group of AOs than those with a relationship to someone with problem gambling only. As such experiencing financial problems may potentially be an early driver of low mental wellbeing among AOs, before further gambling harms progress, with financial problems often a key driver of more psychosocial harms which may then take precedence (Dowling et al., 2021; Holdsworth et al., 2013; Subramaniam et al., 2017).

#### Implications for Policies and Support

The current study adds to the understanding that gambling-related harms impact a far larger proportion of the population than only an individual who gambles at the problem gambling level. Therefore, this study’s findings give support to public health policies that aim to prevent gambling harms across the spectrum of gambling risk levels—which may in turn prevent harms at individual, relationship, and societal levels. Policies that limit susceptibility to financial problems from gambling (such as affordability checks) may prove promising in protecting the health and wellbeing of people who gamble and AOs. Further, policies which aim to reduce the impacts of environmental factors which encourage engagement in multiple health risk behaviours are likely to have health benefits at the population level.

Previous research has found that AOs utilise a range of coping strategies before seeking other forms of support and that this is likely due to barriers, such as stigma and not being aware of what support is on offer, to seeking help from services, including those which offer informal support (Banks et al., 2018; Dowling et al., 2021). Therefore, improving public health messaging to increase awareness of gambling-related harms, what support is available, and encouraging AOs to seek support is essential to meeting the health and wellbeing needs of AOs. Support for AOs should be easily accessible regardless of the risk level of the other individual’s gambling, or the AO’s relationship to them, and when presenting to services the needs of AOs need to be assessed and managed holistically, including their own gambling and other health risk behaviours.

While gambling-related harms are generally significant before AOs will seek support, they may present earlier at services for other associated problems, such as financial problems or low mental wellbeing, this may then provide opportunities to screen for gambling-related harms and identify any relevant support needs (Butler et al., 2019). To address gambling-related harms it is critical that such opportunities are not missed, therefore routine enquiry into gambling harms (originating from both the individual’s own gambling behaviours and their relationships with others) in the context of presentations for factors such as financial problems or low mental wellbeing, may improve identification of those impacted by gambling harms.

### Limitations

The findings in the current study must be considered in light of the following limitations:

The cross-sectional nature of this study means that any assessment of causality of the associations shown is not possible. It cannot be determined whether being an AO precedes an individual’s own risky gambling behaviours and low wellbeing or vice versa.

Harms may vary between different groups of AOs, however, no sub-group analyses were possible due to the relatively small sample size of AOs. Future exploration of experiences of harm and health risk behaviours in different groups of AOs at the sub-problem gambling level remains of importance.

The frequency of the partner or relative’s gambling was not assessed, and while it is hoped that our measure of ‘gambling regularly in the past 12 months’ will have included a range of partner or relative gambling behaviours (particularly including those at a sub-problem gambling level), it may be that only partners or relatives with a problem gambling were included. However, the higher prevalence of AOs in the current study (11.0%) compared to a previous UK-based study (6.5%; Gunstone et al., 2021), suggests that a wider group may have been included. In future research, if a partner or relative’s PGSI score were to be available this would be advantageous.

## Conclusion

While gambling-related harms are increasingly being considered as a public health issue, health and social harms to AOs remain poorly understood and addressed. Most research on AOs has not taken into account harms that can occur at the sub-problem gambling level, and such evidence is particularly limited from a British perspective. This study aimed to address this gap by utilising a measure for identifying AOs in a general population which did not depend solely on the AO having a relationship with someone who gambles at the problem gambling level. This study indicated that there are associations between being an AO, their own risky gambling behaviours, financial problems, and poorer wellbeing. Support for AOs should therefore be more widely available, and not limited to those with a relationship with someone who gambles at the problem gambling level. Further, support for AOs should aim to address their needs holistically to have the greatest impact on wellbeing.

## Supplementary Information

Below is the link to the electronic supplementary material.

Supplementary file1 (DOCX 38 KB)

## Declarations

### Data availability statement

The study materials and data are available from the corresponding author upon reasonable request.
