# Supplementary material for: Harms from Other People’s Gambling: Associations with an Individual’s Own Gambling Behaviours, Health Risk Behaviours, Financial Problems, General Health, and Mental Wellbeing
Source: J Gambl Stud. 2024 Mar 15;40(3):1–15. doi: 10.1007/s10899-024-10291-w (PMC11390759; doi:10.1007/s10899-024-10291-w)
Supplement: Supplementary file 1 — Supplementary file1 (DOCX 38 KB) [file 10899_2024_10291_MOESM1_ESM.docx]

**Multivariate relationships between health risk behaviours, financial harm, general health, and mental wellbeing, and affected other status**

**Supplementary table 1 relationship between poor diet and affected other status:**

|  | **AOR (95% CI)** | **Significance (*p*)** |
| --- | --- | --- |
| **Gender** |  |  |
| Male (ref) | - | - |
| Female | 0.52 (0.32-0.85) | 0.009 |
| **Age** |  |  |
| 16-34 (ref) | - | - |
| 35-54 | 0.66 (0.35-1.24) | 0.194 |
| 55+ | 0.41 (0.22-0.77) | 0.006 |
| **Income level** |  |  |
| <£20,000 (ref) | - | - |
| £20,000-79,999 | 0.88 (0.37-2.06) | 0.763 |
| £80,000+ | 0.39 (0.15-1.04) | 0.060 |
| **Gambling risk level** |  |  |
| Non-problem gambling (ref) | - | - |
| At-risk/problem gambling | 2.19 (1.02-4.72) | 0.046 |
| **Affected other status** |  |  |
| Non-affected others (ref) | - | - |
| Affected others | 1.48 (0.75-2.91) | 0.255 |

**Supplementary table 2 relationship between low physical activity and affected other status:**

|  | **AOR (95% CI)** | **Significance (*p*)** |
| --- | --- | --- |
| **Gender** |  |  |
| Male (ref) | - | - |
| Female | 1.44 (1.09-1.91) | 0.010 |
| **Age** |  |  |
| 16-34 (ref) | - | - |
| 35-54 | 0.97 (0.64-1.46) | 0.877 |
| 55+ | 0.79 (0.53-1.17) | 0.230 |
| **Income level** |  |  |
| <£20,000 (ref) | - | - |
| £20,000-79,999 | 1.12 (0.67-1.87) | 0.676 |
| £80,000+ | 0.83 (0.48-1.45) | 0.518 |
| **Gambling risk level** |  |  |
| Non-problem gambling (ref) | - | - |
| At-risk/problem gambling | 1.07 (0.59-1.95) | 0.817 |
| **Affected other status** |  |  |
| Non-affected others (ref) | - | - |
| Affected others | 1.29 (0.84-1.98) | 0.242 |

**Supplementary table 3 relationship between daily smoking and affected other status:**

|  | **AOR (95% CI)** | **Significance (*p*)** |
| --- | --- | --- |
| **Gender** |  |  |
| Male (ref) | - | - |
| Female | 0.93 (0.53-1.63) | 0.790 |
| **Age** |  |  |
| 16-34 (ref) | - | - |
| 35-54 | 0.77 (0.39-1.55) | 0.467 |
| 55+ | 0.25 (0.12-0.53) | <0.001 |
| **Income level** |  |  |
| <£20,000 (ref) | - | - |
| £20,000-79,999 | 0.25 (0.12-0.54) | <0.001 |
| £80,000+ | 0.11 (0.04-0.28) | <0.001 |
| **Gambling risk level** |  |  |
| Non-problem gambling (ref) | - | - |
| At-risk/problem gambling | 1.58 (0.65-3.89) | 0.315 |
| **Affected other status** |  |  |
| Non-affected others (ref) | - | - |
| Affected others | 1.55 (0.73-3.27) | 0.253 |

**Supplementary table 4 relationship between binge drinking and affected other status:**

|  | **AOR (95% CI)** | **Significance (*p*)** |
| --- | --- | --- |
| **Gender** |  |  |
| Male (ref) | - | - |
| Female | 0.38 (0.26-0.56) | <0.001 |
| **Age** |  |  |
| 16-34 (ref) | - | - |
| 35-54 | 2.34 (1.27-4.33) | 0.007 |
| 55+ | 1.29 (0.70-2.38) | 0.419 |
| **Income level** |  |  |
| <£20,000 (ref) | - | - |
| £20,000-79,999 | 1.16 (0.52-2.61) | 0.713 |
| £80,000+ | 0.98 (0.42-2.29) | 0.965 |
| **Gambling risk level** |  |  |
| Non-problem gambling (ref) | - | - |
| At-risk/problem gambling | 1.53 (0.72-3.23) | 0.265 |
| **Affected other status** |  |  |
| Non-affected others (ref) | - | - |
| Affected others | 1.65 (0.96-2.85) | 0.070 |

**Supplementary table 5 relationship between engaging in 2 or more health risk behaviours and affected other status:**

|  | **AOR (95% CI)** | **Significance (*p*)** |
| --- | --- | --- |
| **Gender** |  |  |
| Male (ref) | - | - |
| Female | 0.63 (0.41-0.99) | 0.042 |
| **Age** |  |  |
| 16-34 (ref) | - | - |
| 35-54 | 1.40 (0.74-2.64) | 0.300 |
| 55+ | 0.73 (0.39-1.39) | 0.339 |
| **Income level** |  |  |
| <£20,000 (ref) | - | - |
| £20,000-79,999 | 0.68 (0.31-1.51) | 0.346 |
| £80,000+ | 0.39 (0.16-0.92) | 0.032 |
| **Gambling risk level** |  |  |
| Non-problem gambling (ref) | - | - |
| At-risk/problem gambling | 2.42 (1.16-5.05) | 0.018 |
| **Affected other status** |  |  |
| Non-affected others (ref) | - | - |
| Affected others | 1.75 (0.97-3.17) | 0.065 |

**Supplementary table 6 relationship between experiencing financial problems and affected other status:**

|  | **AOR (95% CI)** | **Significance (*p*)** |
| --- | --- | --- |
| **Gender** |  |  |
| Male (ref) | - | - |
| Female | 1.45 (0.84-2.49) | 0.178 |
| **Age** |  |  |
| 16-34 (ref) | - | - |
| 35-54 | 1.36 (0.74-2.49) | 0.326 |
| 55+ | 0.14 (0.06-0.32) | <0.001 |
| **Income level** |  |  |
| <£20,000 (ref) | - | - |
| £20,000-79,999 | 0.48 (0.20-1.15) | 0.101 |
| £80,000+ | 0.12 (0.04-0.33) | <0.001 |
| **Gambling risk level** |  |  |
| Non-problem gambling (ref) | - | - |
| At-risk/problem gambling | 3.34 (1.60-6.97) | 0.001 |
| **Affected other status** |  |  |
| Non-affected others (ref) | - | - |
| Affected others | 2.12 (1.10-4.07) | 0.025 |

**Supplementary table 7 relationship between poor general health and affected other status:**

|  | **Model 1** | | **Model 2 *(including health risk behaviours)** | |
| --- | --- | --- | --- | --- |
|  | **AOR (95% CI)** | **Significance (*p*)** | **AOR (95% CI)** | **Significance (*p*)** |
| **Gender** |  |  |  |  |
| Male (ref) | - | - | - | - |
| Female | 0.99 (0.66-1.47) | 0.942 | 0.63 (0.41-0.99) | 0.042 |
| **Age** |  |  |  |  |
| 16-34 (ref) | - | - | - | - |
| 35-54 | 0.93 (0.53-1.62) | 0.791 | 1.40 (0.74-2.64) | 0.300 |
| 55+ | 0.56 (0.32-0.96) | 0.035 | 0.73 (0.39-1.39) | 0.339 |
| **Income level** |  |  |  |  |
| <£20,000 (ref) | - | - | - | - |
| £20,000-79,999 | 0.26 (0.15-0.46) | <0.001 | 0.68 (0.31-1.51) | 0.346 |
| £80,000+ | 0.14 (0.07-0.27) | <0.001 | 0.39 (0.16-0.92) | 0.032 |
| **Diet** |  |  |  |  |
| Not poor diet (ref) | - | - | - | - |
| Poor diet | - | - | 1.51 (0.73-3.14) | 0.269 |
| **Physical activity** |  |  |  |  |
| Enough physical activity (ref) | - | - | - | - |
| Low physical activity | - | - | 2.30 (1.42-3.71) | <0.001 |
| **Smoking** |  |  |  |  |
| Not daily smoking (ref) | - | - | - | - |
| Daily smoking | - | - | 1.91 (0.88-4.17) | 0.104 |
| **Alcohol drinking** |  |  |  |  |
| Not binge drinking (ref) | - | - | - | - |
| Binge drinking | - | - | 0.72 (0.36-1.44) | 0.350 |
| **Gambling risk level** |  |  |  |  |
| Non-problem gambling (ref) | - | - | - | - |
| At-risk/problem gambling | - | - | 1.11 (0.44-2.80) | 0.825 |
| **Affected other status** |  |  |  |  |
| Non-affected others (ref) | - | - | - | - |
| ted others | 1.82 (1.06-3.13) | 0.030 | 1.85 (0.99-3.47) | 0.055 |

**Supplementary table 8 relationship between low mental wellbeing and affected other status:**

|  | **Model 1** | | **Model 2 *(including health risk behaviours)** | | **Model 2 *(including financial harms)** | |
| --- | --- | --- | --- | --- | --- | --- |
|  | **AOR (95% CI)** | **Significance (*p*)** | **AOR (95% CI)** | **Significance (*p*)** | **AOR (95% CI)** | **Significance (*p*)** |
| **Gender** |  |  |  |  |  |  |
| Male (ref) | - | - | - | - | - | - |
| Female | 1.06 (0.74-1.51) | 0.751 | 1.17 (0.78-1.75) | 0.457 | 1.11 (0.73-1.67) | 0.636 |
| **Age** |  |  |  |  |  |  |
| 16-34 (ref) | - | - | - | - | - | - |
| 35-54 | 1.15 (0.70-1.87) | 0.583 | 1.12 (0.64-1.95) | 0.699 | 1.03 (0.58-1.83) | 0.911 |
| 55+ | 0.51 (0.31-0.84) | 0.008 | 0.62 (0.35-1.10) | 0.101 | 0.70 (0.39-1.25) | 0.224 |
| **Income level** |  |  |  |  |  |  |
| <£20,000 (ref) | - | - | - | - | - | - |
| £20,000-79,999 | 0.52 (0.29-0.93) | 0.028 | 0.75 (0.35-1.61) | 0.459 | 0.78 (0.36-1.71) | 0.531 |
| £80,000+ | 0.31 (0.16-0.59) | <0.001 | 0.52 (0.23-1.19) | 0.124 | 0.62 (0.27-1.45) | 0.269 |
| **Diet** |  |  |  |  |  |  |
| Not a poor diet (ref) | - | - | - | - | - | - |
| Poor diet | - | - | 1.54 (0.79-3.00) | 0.209 | 1.34 (0.67-2.69) | 0.409 |
| **Physical activity** |  |  |  |  |  |  |
| Enough physical activity (ref) | - | - | - | - | - | - |
| Low physical activity | - | - | 1.02 (0.67-1.57) | 0.924 | 1.10 (0.71-1.70) | 0.666 |
| **Smoking** |  |  |  |  |  |  |
| Not daily smoking (ref) | - | - | - | - | - | - |
| Daily smoking | - | - | 1.21 (0.57-2.59) | 0.620 | 0.97 (0.43-2.16) | 0.936 |
| **Alcohol drinking** |  |  |  |  |  |  |
| Not binge drinking (ref) | - | - | - | - | - | - |
| Binge drinking | - | - | 1.08 (0.64-1.84) | 0.763 | 1.15 (0.67-1.95) | 0.619 |
| **Gambling risk level** |  |  |  |  |  |  |
| Non-problem gambling (ref) | - | - | - | - | - | - |
| At-risk/problem gambling | - | - | 1.66 (0.80-3.43) | 0.175 | 1.32 (0.60-2.88) | 0.487 |
| **Financial problems** |  |  |  |  |  |  |
| No financial problems (ref) | - | - | - | - | - | - |
| Experiences financial problems | - | - | - | - | 3.82 (2.07-7.04) | <0.001 |
| **Affected other status** |  |  |  |  |  |  |
| Non-affected others (ref) | - | - | - | - | - | - |
| Affected others | 1.70 (1.04-2.77) | 0.035 | 1.75 (1.02-2.99) | 0.042 | 1.65 (0.95-2.87) | 0.076 |
